# Cervical Cytopathology in a Population of HIV-Positive and HIV-Negative Women

**DOI:** 10.1155/2012/869758

**Published:** 2012-07-24

**Authors:** Patrícia Abreu Pinheiro de Lemos, Marco Túlio Antonio García-Zapata, Suelene Brito do Nascimento Tavares

**Affiliations:** ^1^Institute of Tropical Pathology and Public Health, Federal University of Goiás, 74645-050 Goiânia, Go, Brazil; ^2^Cytology Department, Romulo Rocha Clinical Analysis, Federal University of Goiás, 74645-220 Goiânia, Go, Brazil

## Abstract

The association between abnormal cervical cytology and HIV infection status in women was evaluated to correlate with CD4 cell count and viral load in HIV-positive patients with the presence of low-grade (LSIL) and high-grade squamous intraepithelial lesion (HSIL). Cervical samples were collected at the Tropical Disease Hospital, Maternal and Child Healthcare Hospital and at the *Nascer Cidadão* Maternity Hospital in Goiânia, Goiás, Brazil. An Ayre's spatula was used to collect samples from the ectocervix and a cytology brush to collect samples from the endocervix. Of a total of 237 women, 125 were HIV positive and 112 were HIV negative. Abnormal cytology (*n* = 21; 8.9%) was more common in the HIV positive group (*n* = 15; 12.1%) compared to the HIV-negative group (*n* = 6; 5.4%) (*P* = 0.05). Cytological abnormalities were not found to be associated with immunosuppression, defined as CD4 count < 200 cells/mm^3^. A higher frequency was found between higher viral loads (>10,000/mm^3^) and the presence of abnormal cytology. Pregnant women, irrespective of whether they were HIV positive or negative, were less likely to have lesions compared to the nonpregnant women in the same groups. The higher frequency of abnormal findings in Papanicolaou cytology in HIV-positive women with higher viral loads suggests the association between preinvasive cervical lesions and human immunodeficiency.

## 1. Introduction

Around 33.4 million individuals are infected by the human immunodeficiency virus (HIV) worldwide. According to the World Health Organization (WHO), the overall number of new cases of HIV infection has decreased 17% since 2001 [1]. There are currently two million HIV-positive individuals in Latin America, an increase of 25% in relation to 2001. In Brazil, statistics indicate a total of around 1,230,000 HIV-positive individuals [2]. In the municipality of Goiânia in the state of Goiás, where the population is calculated at 1,281,793 inhabitants, around 4,085 cases of the disease have been registered since 1984 [3]. As discussed by Spitzer [4], the importance of the Papanicolaou smear in detecting preinvasive cervical lesions and this test has been considered routine for cervical screening for various decades and has reduced mortality from cervical cancer by 80%. 

An association between HIV and cervical cancer was first suspected in 1998. Nicol et al. [5] demonstrated a significant change in the cell cycle through the expression of certain regulatory proteins in cervical smears from patients coinfected with human papillomavirus (HPV) and HIV compared to a control group of HPV-positive/HIV-negative patients. The US Centers for Disease Control and Prevention (CDC) included high-grade squamous intraepithelial lesions (HSIL) in category B, which characterizes early symptomatic HIV infection [6]. 

Auge et al. [7] reported a statistically significant difference between HIV-positive women and a control group. Likewise, Mbu et al. [6] confirmed the presence of low-grade squamous intraepithelial lesions (LSILs) and HSIL in pregnant women. Moodley and Garib [8] performed biopsies and reported a greater prevalence of LSIL and HSIL in HIV-positive women; however, the difference between the groups was only significant when all grades were taken into consideration, including those showing only the cytopathic effects of HPV. De Souza et al. [9] associated HPV types and the presence of cervical lesions and found that eight types of high-risk HPV were associated with LSIL, while seven resulted in HSIL. Only one low-risk HPV type was found to be associated with LSIL. Immunosuppression status has been suggested by some authors as constituting a factor that contributes towards the development of preinvasive lesions [6, 10, 11]. Therefore, the objective of this study was to evaluate the association between abnormal cervical cytology findings and HIV infection status in women and to relate the CD4 count and viral load of these HIV-positive patients with the presence of LSIL and HSIL. 

## 2. Material and Methods

The present study was conducted in three large hospitals in Goiânia, Goiás, Brazil. The Tropical Disease Hospital, a hospital specialized in infectious and contagious diseases situated in the mid-western region of the country, has been in operation since 1980. It is a tertiary care hospital that forms part of the Brazilian National Health Service (SUS) and is a national referral center. The Maternal and Child Healthcare Hospital is a state hospital that also forms part of the SUS network. It was inaugurated in 1973 and is a referral hospital in pediatrics, gynecology, and obstetrics. The *Nascer Cidadão *Maternity Hospital is a municipal hospital within the SUS network and is maintained by the Goiânia city council. 

This study was conducted in accordance with the recommendations of the Declaration of Helsinki [12] and in compliance with Resolution 196/96 of the Brazilian Ministry of Health's National Health Council. The internal review boards of the hospitals in which the study was conducted approved the protocol prior to commencement, and all the patients signed an informed consent form. 

A total of 237 women were recruited to the study between August 2005 and November 2006, 125 of whom were confirmed HIV-positive and 112 HIV-negative. Of these, 39 patients were pregnant, 31 in the HIV-positive group, and 8 in the HIV-negative group. The clinical and demographic data of these patients were collected by the principal investigator using a specific questionnaire applied during a gynecological consultation. All the patients were included in the study. The cervical samples were obtained in the three participating hospitals, where they were analyzed by the investigator. 

The study inclusion criteria consisted of sexually active women of reproductive age; if pregnant, at an adequate stage in the pregnancy to permit cervical sampling; women who voluntarily agreed to participate in the study after having been informed of the procedures and risks involved, and who signed an informed consent form in the presence of a witness. The above-mentioned criteria applied to both HIV-positive and -negative women; however, in the case of HIV-positive women, additional inclusion criteria required a confirmed diagnosis of HIV infection (ELISA and Western blot) and the patients' awareness of their primary conditions. 

Cervical samples were obtained by the investigator and by collaborators in the participating hospitals, and the clinical data were recorded. An Ayres spatula was used to obtain material from the ectocervix and a cytology brush for samples from the endocervix. Samples were distributed evenly on glass slides, duly identified, and fixed using a mixture of polyethylene glycol and 70% ethanol to prevent drying. The smears were then submitted to Papanicolaou staining. 

The data collected in this study were stored and analyzed using the EpiInfo software program, version 3.5. Statistical significance was defined as *P* < 0.05. 

## 3. Results

Of the 237 women studied, 125 were HIV positive and 112 HIV negative. With respect to cytology, 15 (12.1%) of the HIV-positive women and 6 (5.4%) of the HIV-negative women tested positive for abnormal cytology (*P *= 0.113) (Table 1).

A greater frequency of intraepithelial lesions was found in the HIV-positive compared to the HIV-negative women. Eleven cases of LSIL were found, 8 in the HIV-positive and 3 in the HIV-negative women (6.4% versus 2.7%, resp.). Six cases of atypical squamous cells of undetermined significance (ASC-US) were found in the HIV-positive women (4.8%), whereas, in the HIV-negative participants, only 2.7% were found to have ASC-US (3 cases). Only one case of HSIL was found in all the smears analyzed, and this patient was HIV positive (Table 1). 

When the presence of LSIL was correlated with CD4 cell count in the HIV-positive patients, these abnormalities were found to be more common in women considered immunosufficient (>200 cells/*μ*l). Abnormal cytology was not correlated with immunosuppression (*P* > 0.05 or *P* = 1, Table 2).

With respect to viral load, the highest frequency of abnormal cytology was found in patients with a viral load >10,000 copies/ml. This association was not, however, statistically significant (*P *> 0.05 or *P* = 1) (Table 3). 

Abnormal cytology was not correlated with the presence of pregnancy (*P* > 0.05 or *P* = 0.434). LSIL was found in 9 HIV-positive women; of these, 2 were pregnant (25.0%) and 7 were not pregnant (77.8%). LSIL was found in 3 HIV-negative women, all of whom were in the nonpregnant group (100.0%). A single case of HSIL was detected in the entire study population and referred to a nonpregnant HIV-positive woman (Table 4). 

## 4. Discussion

The highest frequency of abnormal cytology findings occurred in the group of HIV-positive women. However not statistically significant the highes frequency found in this study is in agreement with studies conducted by Auge et al. [7] and Moodley and Garib [8]. The results likewise, Moodley and Garib [8] reported a statistically significant difference with respect to the human immunodeficiency virus when smears with only the cytopathic effects of HPV were considered. 

In the present study, the presence of lesions was not associated with immunosuppression, bearing in mind that the majority of patients in the HIV-positive and HIV-negative groups had CD4 cell counts >200/mm3. This finding is in accordance with the findings of Zimmermman et al. [10], who reported no difference in CD4+ T-lymphocyte count in the presence or absence of LSIL or HSIL. Nevertheless, Coelho et al. [11] evaluated 115 HIV-positive women and reported higher CD4 counts (201–499) associated with LSIL and HSIL; however, these results were also not statistically significant. Adam et al. [13] reported that cytological abnormalities found following removal of the transformation zone, in addition to being 8 times more frequent in HIV-positive patients, were also twice as common in women with a CD4 cell count <200/mm3.

The finding of higher viral counts (>10,000 copies/mm3) when lesions are present is in agreement with the results reported by Massad et al. [14] and emphasizes the close relationship between HIV/HPV and a consequent cytological abnormality. 

The finding that the concomitant presence of LSIL and HIV was less frequent in the pregnant women compared to the women in the nonpregnant group (25% versus 77.8%) contradicts the data reported by Mbu et al. [6] whose study conducted in Cameroon (Africa) reported rates of 18.2% in HIV-positive pregnant women compared to 4.4% in the nonpregnant control group. The difference in the results of the present study may be due to the fact that the pregnant HIV-positive women receiving care at the Maternal and Child Healthcare Maternity Hospital were undergoing regular prenatal followup, meaning that any cervical lesions that may have been present would have already been treated. Nevertheless, it is important to emphasize that Marana et al. [15] reported high recurrence rates of low- and high-grade lesions in a group of 12 seropositive pregnant women following conventional treatment and found a statistically significant difference between this group and a control group. 

The finding of a greater frequency of positive Papanicolaou smears associated with a higher viral load in HIV-positive women suggests a possible association between preinvasive cervical lesions and human immunodeficiency. 

## Figures and Tables

**Table 1:** Association between HIV status and cytology findings.

HIV status	Unknown	Negative	Positive	ASC-US	ASC-H	LSIL	HSIL	Total
Positive (%)	1 (0.8)	109 (87.2)	15 (12.1)	6 (4.8)	0 (0.0)	8 (6.4)	1 (0.8)	125 (100.0)
Negative %	0.0 (0.0)	106 (94.6)	6 (5.4)	3 (2.7)	0 (0.0)	3 (2.7)	0 (0.0)	112 (100.0)
Total (%)	1 (0.4)	215 (90.7)	21 (8.9)	9 (3.8)	0 (0.0)	11 (4.6)	1 (0.4)	237 (100.0)

Unknown: results of cytology unknown.

ASC-US: atypical squamous cells of unknown significance.

ASC-H: atypical squamous cells, cannot exclude a high-grade squamous intraepithelial lesion (HSIL).

LSIL: low-grade squamous intraepithelial lesion, according to the Bethesda system.

HSIL: high-grade squamous intraepithelial lesion, according to the Bethesda system.

**Table 2:** Frequency of abnormal cytology correlated with immunological status in the HIV-positive women.

Abnormal cytology	Immunodeficiency (<200 cels. CD4/mm^3^)		Immunosufficiency (>200 cels. CD4/mm^3^)		Total	
	*N*	%	*N*	%	*N*	%
ASC-US	2	33	4	67	6	100
LSIL	2	25	6	75	8	100
HSIL	0	0	1	100	1	100

Unknown: results of cytology unknown.

ASC-US: atypical squamous cells of unknown significance.

ASC-H: atypical squamous cells, cannot exclude a high-grade squamous intraepithelial lesion (HSIL).

LSIL: low-grade squamous intraepithelial lesion, according to the Bethesda system.

HSIL: high-grade squamous intraepithelial lesion, according to the Bethesda system.

**Table 3:** Frequency of abnormal cytology correlated with viral load in HIV-positive women.

Preinvasive lesions of the cervix	Below the detection limit		<1.000 copies/mm^3^		>10.000 copies/mm^3^		Total	
	*N*	%	*N*	%	*N*	%	*N*	%
ASC-US	3	50	0		3	50	6	100
LSIL	1	12	2	25	5	63	8	100
HSIL	0		0		0		0	100

Unknown: results of cytology unknown.

ASC-US: atypical squamous cells of unknown significance.

ASC-H: atypical squamous cells, cannot exclude a high-grade squamous intraepithelial lesion (HSIL).

LSIL: low-grade squamous intraepithelial lesion, according to the Bethesda system.

HSIL: high-grade squamous intraepithelial lesion, according to the Bethesda system.

**Table 4:** Frequency of abnormal cytology in HIV+ and HIV− women correlated with pregnancy.

Abnormal cytology	HIV + women				HIV− women			
	Pregnancy				Pregnancy			
		Yes	No	Total		Yes	No	Total
ASC-US	Present	0	5 (100%)	5 (100%)	Present	0	2 (100%)	2 (100%)
	Absent	31 (26%)	89 (74%)	120 (100%)	Absent	8 (7%)	102 (93%)	110 (100%)
LSIL	Present	2 (22%)	7 (78%)	9 (100%)	Present	0	3 (100%)	3 (100%)
	Absent	29 (25%)	87 (75%)	116 (100%)	Absent	8 (7%)	101 (93%)	109 (100%)
HSIL	Present	0	1 (100%)	1 (100%)	Present	0	0	
	Absent	31 (25%)	94 (75%)	125 (100%)	Absent	8 (7%)	104 (93%)	112 (100%)

Unknown: results of cytology unknown.

ASC-US: atypical squamous cells of unknown significance.

ASC-H: atypical squamous cells, cannot exclude a high-grade squamous intraepithelial lesion (HSIL).

LSIL: low-grade squamous intraepithelial lesion, according to the Bethesda system.

HSIL: high-grade squamous intraepithelial lesion, according to the Bethesda system.
